# Extensive heterotopic pancreas in a rare site: A case report and a review of literature

**DOI:** 10.1097/MD.0000000000032241

**Published:** 2023-03-03

**Authors:** Xiaohan Zhang, Lihua Peng, Zikai Wang, Fei Pan, Rongrong Ren, Yan Li, Xiuli Zhang

**Affiliations:** a Department of Gastroenterology and Hepatology, The First Medical Center, Chinese PLA General Hospital, Beijing, China; b School of Medicine, Nankai University, Tianjin, China.

**Keywords:** angular notch, diagnosis, endoscopy, heterotopic pancreas, incision biopsy

## Abstract

Heterotopic pancreas is a pancreatic tissue that occurs outside the normal anatomical site, the most common site is antrum. Due to the lack of specific imaging and endoscopic signs, heterotopic pancreas especially those occurring in the rare site, are often misdiagnosed, and leading to unnecessary surgical treatment. Endoscopic incisional biopsy and endoscopic ultrasound-guided fine-needle aspiration are effective means for diagnosing heterotopic pancreas. We reports a case of extensive heterotopic pancreas in a rare site, which was finally diagnosed by this way.

**Patient concerns:**

A 62-year-old man was admitted due to the presence of an angular notch lesion, which was suspected as gastric cancer before. He denied any history of tumor or gastric disease.

**Diagnoses:**

No abnormality was found in the physical examination and laboratory testing after admission. Computed tomography showed localized thickening of the gastric wall measuring 30 mm in the long diameter. Gastroscope revealed a nodular-like submucosal protuberance at the angular notch with size of about 3*4 cm. Ultrasonic gastroscope showed that the lesion was located in the submucosa. The lesion exhibited mixed echogenicity. The diagnosis can not be identified.

**Interventions:**

2 times of incision biopsy were performed to make a clear diagnosis. Finally, appropriate tissue specimens were obtained for pathology testing.

**Outcomes:**

The patient was diagnosed as heterotopic pancreas according to pathology. He was recommended to undergo observation and regular follow-ups rather than surgery. Then he was discharged home with no discomfort.

**Lessons:**

Heterotopic pancreas occurring in the angular notch is extremely rare, the site is scarcely reported in the relevant literature. Therefore, it is easy to be misdiagnosed. In the cases of an vague diagnosis, endoscopic incisional biopsy or endoscopic ultrasound-guided fine-needle aspiration may be a good choice.

## 1. Introduction

Heterotopic pancreas is a congenital abnormality, first reported in 1727 by Jean–Schultz.^[1]^ Its specific pathogenesis remains unclear. Because most heterotopic pancreas does not cause clinical symptoms, it is often discovered incidentally. According to autopsy results, the estimated incidence is 0.6% to 14.0%.^[2]^ Although the rapid advances of imaging and endoscopic procedures have provided various means for diagnosing heterotopic pancreas, the diagnostic accuracy is still limited by the nonspecific signs and the uncertain biopsy site. Identifying heterotopic pancreas from other subepithelial masses and determining the appropriate treatment for patients pose a challenge for clinicians. This article reports a case of extensive submucosal mass occurring in the angular notch, which was suspected as gastric cancer on contrast-enhanced computed tomography (CT). It was finally diagnosed as the heterotopic pancreas in a rare site by incision biopsies, avoiding unnecessary surgical resection. And the site of occurrence is scarcely reported.

## 2. Case report

A 62-year-old man was admitted due to an angular notch lesion on November 1, 2021, which was detected incidentally during CT and endoscopic examinations. In May, 2021, the patient had persistent pain in the right lumbar region without an obvious cause, and the specific site could not be clearly described. On October 10, 2021, the patient went to a local hospital for a complete examination due to aggravated pain. The contrast-enhanced CT of the upper abdomen revealed the thickened gastric wall at the lesser curvature of the stomach, and the diagnosis of the malignant lesion could not be excluded. The gastroscope revealed chronic atrophic gastritis with erosion and a hump-like protuberance at the angular notch. The lesion is about 3*4cm in size, with mild erosion and congestion locally. Pathology: Chronic atrophic gastritis with moderate intestinal metaplasia without tumor cells. The patient had a history of hypertension, extrasystole, appendectomy, and right clavicle fracture and right rib fracture.

**Figure 1.**
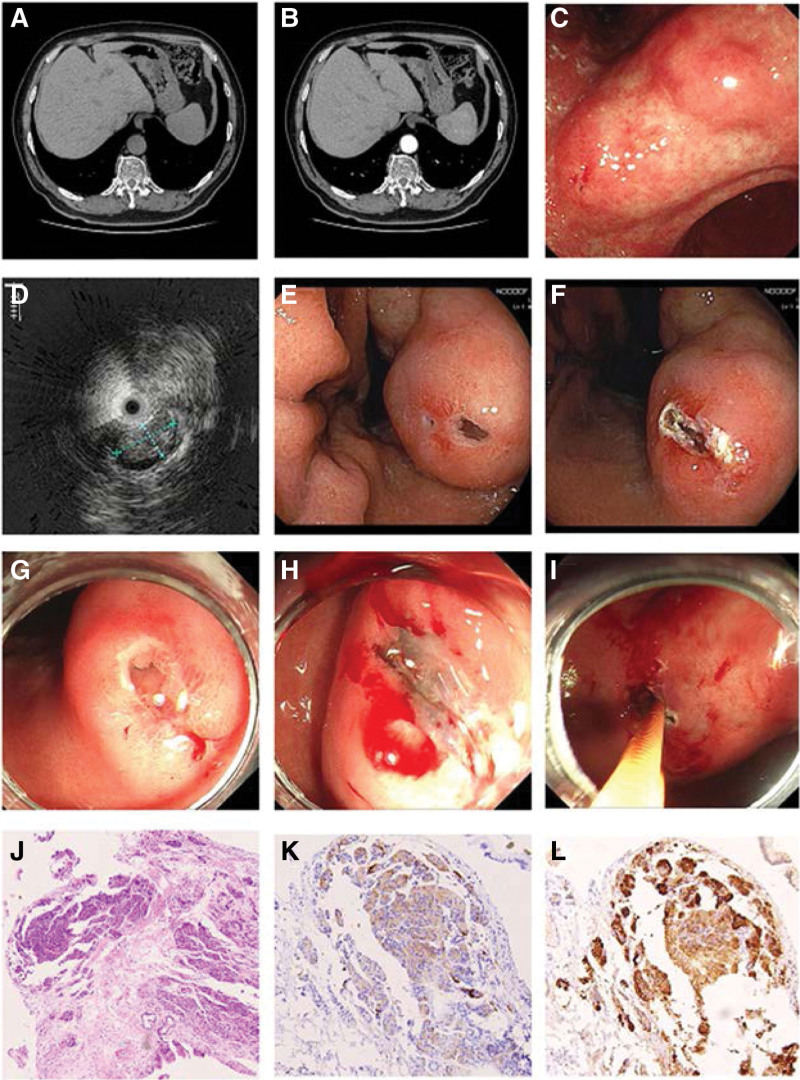
Clinical data of the extensive HP in the angular notch. (A), (B) Plain and enhanced CT images. An oval lesion with uneven density and cystic structure can be observed and the lesions showed slight uneven enhancement. (C), (D) Endoscopic and endoscopic ultrasonography manifestations. A nodular-like submucosal mass located in the angular notch near the lesser curvature (C). The lesion exhibited mixed echogenicity with unclear boundary and cystic structure (D). (E), (F) Incision biopsy to the submucosal mass. Sinus tract forming after biopsy could be observed (E). Dual knife was used to expand the incision (F). (G)–(I) The second incision biopsy. The incision of last biopsy can be observed and there seem to be clear secretion at the opening (G). Expand the incision by Dual knife (H). Biopsy forceps reached into to seek deep specimens (I). (J)–(L) Pathological manifestations. Hematoxylin and eosin staining of HP tissues sections (magnification × 10) (J). Immunohistochemistry: Syn weak + (magnification × 20) (K). Immunohistochemistry: Trypsin + (magnification × 20) (L). CT = computed tomography.

No abnormality was found in the physical examination after admission. No obvious abnormalities were found in blood routine examination, urine routine examination, stool routine examination, and blood biochemical examination and tumor markers. The human pepsinogen I was 37.6ng/mL ↓. Abdominal ultrasonography revealed fatty liver and gallstone. Abdominal contrast-enhanced CT showed localized thickening of the gastric wall on the lesser curvature of the stomach near the angular notch with a long diameter of about 30 mm and a maximum thickness of about 20 mm, the internal density of which was uneven. Moreover, the lesion showed slight uneven enhancement after the injection of contrast media (Fig. 1A and B). Gastric cancer was suspected for diagnosis by imaging. Gastroscope performed on November 5 revealed a nodular-like submucosal protuberance at the angular notch near the lesser curvature of stomach, with a smooth surface and size of about 3*4 cm. Erosion after biopsy in the local hospital could be observed. Ultrasonic gastroscope showed that the lesion was located in the submucosa, but its range could not be evaluated. The lesion exhibited mixed echogenicity. Multiple cystic structures could be observed. Part of the muscularis propria was intact (Fig. 1C and D). Incision biopsy to the submucosal mass was performed on November 8, 2021. The sinus tract forming after biopsy in the local hospital could be seen on the gastroscope and it had a cystic texture. A dual knife was used to expand the incision of the sinus tract. Biopsy forceps reached into to seek deep specimens. A total of 5 pieces of biopsy tissue were taken from the interior of the lesion, the texture of which is tough (Fig. 1E and F). Pathology: Chronic inflammation of broken columnar epithelial mucosa, intestinal metaplasia and hyperplasia of some glands, edema of local lamina propria with inflammatory cell infiltration. The tissue obtained for pathological examination was shallow. In order to make a clear diagnosis, an incision biopsy was performed again after 7 days. The incision of the last biopsy could be observed, which was not completely healed. There seemed to be clear secretion at the opening. The lesion was lifted well after multi-point injection. Use the Daul knife to expand the incision of the mucosal opening. Insert the biopsy forceps into the interior and take 1 bottle of specimens from multiple locations. The texture is tough. Slight bleeding could be observed after biopsy, so electrocoagulation was used for hemostasis and the endoscope clips to close the incision (Fig. 1G–I). Pathology: Chronic inflammation of the mucosa. Heterotopic pancreatic tissue could be seen in subepithelial fibrous connective tissue. Immunohistochemistry: pancreatic tissue Trypsin (+), Syn (weak +) (Fig. 1J–L). It was diagnosed as a heterotopic pancreas finally. The patient was recommended to undergo observation and regular follow-ups. Then he was discharged home with no discomfort.

## 3. Discussion

Heterotopic pancreas is a congenital anomaly developed during the development of the pancreas. The heterotopic pancreatic tissue is completely anatomically separated from the normal pancreas tissue, and there is no vascular or ductal continuity between them. It is also known as ectopic pancreas or aberrant pancreas.^[3–5]^ Autopsy findings show that its incidence is between 0.6% to 14.0%,^[2]^ but the clinical incidence rate may be lower than that figure as patients with this disease are usually asymptomatic. Heterotopic pancreas usually occurs in the upper gastrointestinal tract, such as the stomach, duodenum and proximal jejunum. Esophagus, ileum, Michael diverticulum, biliary system, colon, fallopian tube, umbilical cord, mediastinum, lung, spleen, and liver and omentum are rare sites of occurrence.^[3,6]^ Gastric heterotopic pancreas is common in the antrum and the greater curvature. In this case, the heterotopic pancreas occurs in the angular notch near the lesser curvature of stomach with a wide area. In the relevant literature we searched, heterotopic pancreatic occurring in the angular notch is scarcely reported. And such extensive lesion is also rare.

Heterotopic pancreas is usually asymptomatic, so the lesion is discovered incidentally in irrelevant surgery, imaging or endoscopy examination for other diseases in most cases. Making a definitive diagnosis of heterotopic pancreas can be challenging. Many patients are misdiagnosed with other diseases and undergo unnecessary surgery or even extensive resection. A single-center retrospective study revealed that 54.9% of patients with heterotopic pancreas could be misdiagnosed so that the management would be misled.^[7]^ In this case, the patient was also misdiagnosed with gastric cancer by imaging. Therefore, a precise diagnosis is crucial. Heterotopic pancreas appears as round or oval submucosal lesions covered with normal mucosa under endoscopy. Umbilication is the main feature of this disease but is not seen in all cases. The typical signs of heterotopic pancreas on endoscopic ultrasonography include heterogeneous echogenicity, unclear boundary and a location in muscularis mucosa or deeper layer.^[8]^ CT images commonly show an oval mass with a broad base and an endoluminal growth pattern. The cystic area can be seen in some cases. The presence of a central umbilical sign or a duct-like structure is significance indicative for diagnosis.^[9]^ Due to the nonspecific imaging and endoscopic features above, it is hard to identify heterotopic pancreas from other subepithelial masses such as stromal tumors or leiomyomasy by them alone.^[2,10]^ Although some studies have reported that CT combined with endoscopic ultrasonography can improve the diagnostic accuracy,^[11]^ pathology is still the gold standard for diagnosing heterotopic pancreas. However, due to its subepithelial location, appropriate tissue specimens are difficult to obtain by conventional superficial endoscopic biopsies. Incision biopsy on endoscopic or endoscopic ultrasound-guided fine-needle aspiration is more effective for definite diagnosis.^[8,12]^ Attwell, A, et al^[13]^ reviewed 10 patients diagnosed with heterotopic pancreas in a single-center. Fine-needle aspiration was attempted in 9, of whom 7 were successfully diagnosed with heterotopic pancreas. In the other 2 cases, 1 only showed acute inflammatory cell due to insufficient sample, and 1 did not undergo aspiration due to the small size and difficult location. However, those 2 patients were finally diagnosed as heterotopic pancreas through deep biopsy. One other patient was diagnosed after surgical resection. In this study, the accuracy of fine-needle aspiration was 74% (7/9), the preoperative diagnostic rate was 90%. It also indicates that deep incision biopsy can help obtain tissue specimens more directly and can be performed when fine-needle aspiration is difficult to perform or nondiagnostic. Due to the extensive range of the lesion and the formation of sinus tract after biopsy, incision biopsy was chosen to perform in this case to obtain more tissue specimens directly. It helped make the definite diagnosis based on ultrasonic gastroscopy diagnosis, avoiding unnecessary surgery or even an expanded surgical resection on the patient.

Malignant transformation of heterotopic pancreas is a rare event in the clinic, of which incidence is reported to be 0.7% to 1.8%.^[14]^ A retrospective analysis of 54 patients with malignant heterotopic pancreas demonstrates that the incidence is slightly higher in male patients, and it most commonly occurs in the gastric heterotopic pancreas. The pathological type was usually adenocarcinoma.^[15]^ There is no sufficient evidence that cancerous and precancerous lesions are more likely to occur in the heterotopic pancreas,^[3,15]^ but it should still be regarded as a potential source of malignant transformation. Therefore, there is no urgent need to perform surgical intervention for asymptomatic patients with heterotopic pancreas, but periodic follow-up is recommended. Such management can avoid unnecessary surgical resection while ensuring a good prognosis for patients to maximize their benefit.

## Author contributions

**Conceptualization:** Lihua Peng, Xiuli Zhang.

**Investigation:** Rongrong Ren, Yan Li.

**Methodology:** Lihua Peng.

**Supervision:** Xiuli Zhang.

**Writing – original draft:** Xiaohan Zhang.

**Writing – review & editing:** Zikai Wang, Fei Pan.
